# Maintenance of Chronological Aging Features in Culture of Normal Human Dermal Fibroblasts from Old Donors

**DOI:** 10.3390/cells11050858

**Published:** 2022-03-02

**Authors:** Julie Rorteau, Fabien P. Chevalier, Sébastien Bonnet, Théo Barthélemy, Amandine Lopez-Gaydon, Lisa S. Martin, Nicolas Bechetoille, Jérôme Lamartine

**Affiliations:** 1 CNRS UMR 5305, Tissue Biology and Therapeutic Engineering Laboratory (LBTI), 69007 Lyon, France; julie.rorteau@ibcp.fr (J.R.); fabien.chevalier@univ-lyon1.fr (F.P.C.); theo.barthelemy@ibcp.fr (T.B.); lisa.martin@ibcp.fr (L.S.M.); 2 Gattefossé SA, 36 Chemin de Genas, CS 70070, CEDEX, 69804 Saint-Priest, France; sbonnet@gattefosse.com (S.B.); alopezgaydon@gattefosse.com (A.L.-G.); nbechetoille@gattefosse.com (N.B.); 3 Claude Bernard University Lyon 1, 69100 Villeurbanne, France

**Keywords:** fibroblast, aging, population doubling, clonogenicity, mitochondria, extracellular matrix, skin

## Abstract

Chronological aging is defined as a time-dependent decline of tissue homeostasis which severely impacts skin. Understanding the mechanisms of skin aging is an active research area limited by the lack of relevant in vitro models. Being a component of aging, replicative or stress-induced senescence is repeatedly used to mimic skin aging in vitro, thus presenting only a partial view of the complexity of aging. Herein, we aimed to clarify whether primary normal human dermal fibroblasts retained age-related characteristics when cultured in 2D monolayer, and could be used as a relevant model for aging research. We compared three groups of fibroblasts isolated from different aged donors. We observed strongly decreased population doubling capacities, a reduced clonogenic ability, an impairment in extracellular matrix production together with modifications of respiratory metabolism with an increase in age. These disruptions were particularly marked when comparing fibroblasts isolated from old individuals (over 70 years old) to those isolated from young individuals (18–37 years old), while cells from middle-aged donors exhibited an intermediate profile. These alterations of cell features can be related to the signs of dermis aging, thus showing that cultured primary cells indeed retain some characteristics of the original tissue from which they were extracted.

## 1. Introduction

The skin plays a central role in protecting the internal organs from deleterious environmental factors and thus maintains fluid balance and body temperature, both of which are essential for optimal vital functions in humans and animals. As the first physical barrier between the body and the environment, the skin is continuously subjected to external aggressions, from birth to death, including sun exposure, hot and cold temperature variations, atmospheric pollutants, biological assaults or mechanical stimuli. Thus, the skin exhibits an incredible capacity for self-regeneration which is required throughout life. However, as we age, skin homeostasis gradually declines and is accompanied by the appearance of marks such as wrinkles and age spots. Nowadays, slowing down the aging process has emerged as a major medical and societal issue. Therefore, a better understanding of the fundamental mechanisms involved in the loss of skin homeostasis during aging is necessary for developing anti-aging strategies. At the tissue level, aging induces a progressive loss of tissue integrity and impairs cellular functions which leads to the macroscopic manifestations previously mentioned. Some characteristics of skin aging are already well described in the literature. The thinning of epidermis and the defect in barrier function are mainly caused by a progressive slowing of keratinocyte proliferation and differentiation defects [1]. Regarding the dermal compartment, aging induces a disorganization of the extracellular matrix (ECM) with a decrease in the production of collagen and elastin fibers by fibroblasts [2]. This matrix alteration is also due to a deregulation of the expression of proteins involved in ECM remodeling [3]. Since the skin tissue covers the whole body, the aging process is particularly affected by environmental factors. This extrinsic aging has been well described, particularly in the context of photo-aging [4]. Conversely, the consequences at the cellular level of intrinsic, genetically programmed aging are still largely unexplored, with the exception of senescence. Replicative senescence is an irreversible cell cycle arrest, mainly caused by the telomere shortening after successive mitosis. Senescence can also be induced by oxidative stress-related DNA damages or mutations in genes governing cell proliferation [5,6]. Senescence is a form of cell death since affected cells are unable to proliferate even with a sufficient supply of nutrients. Nevertheless, senescent cells remain metabolically active and participate in the dissemination of a deleterious signal in the tissue through a particular secretome called senescent-associated secretory phenotype (SASP), which is composed of cytokines, growth factors and extracellular vesicles integrated by neighboring cells [7,8]. Because cellular senescence is easy to reproduce in vitro by long-term culture, stress exposure or oncogenes overexpression, this model is commonly used to mimic aging and notably dermis aging, which is particularly affected by the presence of senescent cells [9]. However, even if cellular senescence is an important parameter, other elements need to be considered in intrinsic aging phenomenon, such as epigenetic alterations, loss of proteostasis, deregulated nutrient sensing, stem cell exhaustion, mitochondrial dysfunction, altered intercellular communication, genomic instability or telomere attrition [10]. Thus, using in vitro models of aged cells better representative of the reality of aged tissues would facilitate the understanding of chronological aging mechanisms and the identification of biological targets for anti-aging strategies. Therefore, in this study, we wondered whether primary normal human dermal fibroblasts (NHDF) isolated from the skin of young or aged individuals retain the tissue aging signature once maintained in culture. We constituted three groups of age (young, middle-aged and old fibroblasts) and looked at features of fibroblasts behavior potentially impacted by aging such as cell proliferation, energy metabolism and production of ECM. In primary fibroblasts isolated from aged individuals over 70 years old, we observed decreased proliferation capacities, reduced clonogenic abilities, an impaired ECM production and modification of respiratory metabolism, four features of skin aging. Primary fibroblasts isolated from aged skin therefore represent a valuable in vitro model to study some parameters of skin aging.

## 2. Materials and Methods

### 2.1. Cell Culture

Primary NHDF were obtained from healthy breast or abdomen skin biopsies of Caucasian female donors and from eyelid skin biopsies of Caucasian male donors, with their informed consent and in accordance with the ethical guidelines (French Bioethics law of 2004). NHDF were ranked into three age groups: young adult fibroblasts (24.8 ± 8.3 years old; *n* = 5), middle-aged adult fibroblasts (59.8 ± 3.7 years old; *n* = 5) and old adult fibroblasts (74.4 ± 2.3 years old; *n* = 5). Primary fibroblasts from progeria patients were purchased from the Coriell Institute (Camden, NJ, USA). Cells were cultured in DMEM (61965-026, Thermo Fisher Scientific, Waltham, MA, USA), supplemented with 10% foetal bovine serum (FBS) (SH30072, Hyclone, Logan, UT, USA) and 1% penicillin/streptomycin (P/S) (SH30072, Sigma-Aldrich, Saint-Louis, MO, USA) at 37 °C and 5% CO2. The culture medium was changed three times weekly. Cells between passages 5–8 were used in this study. The number of biological replicates of primary NHDF varied between 3 and 5, depending on the experiment, which is indicated in the figure legend. All the characteristics of the biological sources of the cells are listed in the Supplementary Table S2.

### 2.2. SA-β-Galactosidase Staining

The SA-β-gal staining was performed as previously described [11]. Cells were fixed using 4% paraformaldehyde (PFA) for 10 min followed by 3 washes. Next, cells were incubated in staining solution (40 mM citric acid/Na phosphate buffer, 5 mM K_4_[Fe(CN)_6_]3H_2_O, 5 mM K_3_[Fe(CN)_6_], 150 mM NaCl, 2 mM MgCl_2_ and 1 mg/mL X-gal in distilled water for 24 h at 37 °C. Cells were washed three times with distilled water, observed using light microscopy and manually counted. As a positive control, senescence was induced by treatment with 10 µM of etoposide for 24 h.

### 2.3. 53BP1 Immunostaining

Fibroblasts were fixed with 4% PFA for 10 min. Then, cells were permeabilized (0.1% Triton X-100, 0.1 M Glycine) and incubated in blocking buffer (5% goat serum, 2% BSA, 0.1% Triton X-100 and 0.05% Tween-20) for 15 min prior to immunostaining with anti-53BP1 antibody (PA1-46147, ThermoFisher, Waltham, MA, USA). For immunodetection, goat anti-rabbit IgG Alexa Fluor-488 conjugated secondary antibody (ThermoFisher) was incubated for 1 h at RT and nuclei were counterstained with DAPI. The positive cells were counted in three fields per primary fibroblast strains using an Eclipse Ti-E inverted microscope (Nikon France S.A., Champigny sur Marne, France).

### 2.4. Protein Extraction and Immunoblotting

Total proteins were extracted using an RIPA buffer (50 mM Tris-HCl pH = 8, 150 mM NaCl, 1.5 mM KCl, 1% NP-40, 0.1% SDS, 0.5% sodium deoxycholate, 0.1% Triton X-100, 1 mM EDTA) containing protease and a phosphatase inhibitors cocktail (A32961, ThermoFisher). Proteins were quantified using the Pierce BCA Protein Assay Kit (ThermoFisher), loaded on an 10% SDS-polyacrylamide gel and transferred to a PVDF membrane (IPVH85R, Merck, Darmstadt, Germany). The membrane was incubated for 1 h at RT in blocking buffer (TBS-Tween-20 0.1%, 5% milk) and immunoblotted overnight at 4 °C with primary antibodies targeting P16 (#554079, BD Biosciences), P21 (sc-397, Santa Cruz, Dallas, TX, USA) or actin (MAB1501, Merck). After washing, goat anti-mouse IgG HRP-conjugated secondary antibodies (Bio-rad, Hercules, CA, USA) were incubated for 1 h at RT. Proteins were detected using SuperSignal West Pico PLUS Chemiluminescent Substrate (ThermoFisher) and the signal was detected by the Fusion Fx system (Vilber, Marne-la-Vallée, France).

### 2.5. Population Doubling Assay

Cells were plated at 1.8 × 10^4^ cells/cm^2^. At 90% of cell confluence, fibroblasts were trypsinized, numerated and seeded again at constant cell density. This procedure was repeated until cells reached replicative senescence.

### 2.6. Clonogenicity Frequency

Primary fibroblasts were seeded at 16, 8, 4, 2, 1 and 0.5 cells per well, using serial dilutions. A total of 24 replicates were performed for each dilution and the medium was changed every three days. After 15 days, each well was scored as positive (presence of at least one colony) or negative (absence of cells). While a positive well may arise from the presence of one or more cells with clonal expansion ability, negative ones undeniably reveal the absence of fibroblast with clonogenic potential. Therefore, limiting the dilution analysis of unfractionated cell populations results in nonlinear kinetics because of the interaction of several subpopulations of responder cells (with a clonogenic potential), present at distinct cell frequencies. Thus, we estimated the frequency of responder cells by plotting the log of the fraction of negative well against the cell number in the respective replicate cultures. According to Poisson statistics, the most informative value corresponds to a negative fraction of 0.37. Thus, the clonogenicity index was determined as the number of cells/well required to obtain 37% negative wells [12,13].

### 2.7. Proliferation Assay

Cells were plated at 10 × 10^3^ cells/cm^2^ in 96-well plates. Cell-cycle synchronization was performed by starvation with DMEM and 1% FBS 1% P/S overnight. After washing with PBS, the DNA concentration was determined using CyQuantTM Cell Proliferation Assay Kit (C7026, Thermo Fischer Scientific) according to the supplier’s instructions. The fluorescent signal was analyzed using Tecan Infinite M1000 (30034301, Tecan, Männedorf, Switzerland).

### 2.8. Analysis of Energy Metabolism

The oxygen consumption rate (OCR) was measured with a Seahorse XF extracellular flux analyzer according to the manufacturer’s instructions (Agilent, Santa Clara, CA, USA). Primary fibroblasts were seeded at 8 × 10^4^ cells per well in a specialized 24-wells microplate and incubated at 37 °C in 5% CO_2_ overnight. Culture medium was replaced with XF assay medium supplemented with 10 mM glucose (103577-100, Agilent), 1 mM pyruvate (103578-100, Agilent) and 2 mM glutamine (103579-100, Agilent) and cells were incubated in the absence of CO_2_ for 45 min before measurement. OCR was determined before injection of specific metabolic inhibitors and after successively adding 1.5 μM oligomycin, 1 μM FCCP, and 0.5 µM rotenone/antimycin A (Sigma-Aldrich). OCR data were normalized according to fluorescent cell counting using BioTek Cytation (BioTek, Winooski, VT, USA). Wave software was used to analyse Seahorse measurements.

### 2.9. Mitochondria and Reactive Oxygen Species (ROS) Content

Cells were plated at 10 × 10^3^ cells/cm^2^ in 8-well chamber slide (C7182, Thermo Fisher Scientific). At 80% of cell confluence, fibroblasts were incubated 30 min at 37 °C with 100 mM of MitoTracker^TM^ Deep Red FM (M22426, Thermo Fisher Scientific) and 5 µM of CellROX^TM^ Green (C10444, Thermo Fisher Scientific). Cells were fixed using 4% PFA and nuclear staining was performed using ProLong^TM^ Glass Antifade Mountant with NucBlue^TM^ (Thermo Fischer Scientific). Image were visualized using an Eclipse Ti-E inverted microscope (Nikon) and analysed using ImageJ software (version 2.1.0/1.53k).

### 2.10. MMP12 Elastase Activity Assay

Conditioned media from monolayer culture of fibroblasts were harvested and MMP12 enzymatic activity was assessed using SensoLyte 520 MMP12 assay kit (AnaSpec, San Jose, CA, USA) and the microplate reader (Infinite M1000, Tecan, Mannedorf, Switzerland). Results were expressed as RFU (relative fluorescence unit)/DNA amount (ng/mL) ratio.

### 2.11. Elastin, Fibronectin and Collagen Content

Primary antibodies were directed against human elastin (rabbit polyclonal antibody, Novotec, France), human fibronectin (rabbit polyclonal antibody, Sigma, France) and human collagen (rabbit polyclonal antibody, Origene, Rockville, MD, USA). Goat anti-rabbit Alexa Fluor 594 (Invitrogen, Waltham, MA, USA) was used as conjugate for elastin. Fluorescent staining of elastin was observed using Cytation 5 cell imaging multi-mode reader and quantified using Gen5 image software (BioTek, Winooski, VT, USA); the results were expressed as RFU (relative fluorescent unit) % (area of fluorescent regions vs. total area)/nuclei number ratio in cells. Goat anti-rabbit Europium and Delfia^®^ enhancement solutions were used as conjugates (Perkin-Elmer) for fibronectin and collagen. Fluorescent staining of fibronectin and collagen was quantified using a microplate reader (Infinite M1000, TECAN) and results were expressed as RFU (relative fluorescence unit: fluorescent signal) and normalized to the nuclei number.

### 2.12. Gene Expression Analysis and Estimation of mtDNA Content

Total RNA and DNA were isolated using Quick-DNA/RNATM Miniprep kit (Zymo research, Mülhauser, Germany) according to manufacturer’s instruction.

Total mRNA was reverse-transcribed into cDNA using PrimeScriptTM RT reagent kit (Takara, Shiga, Japan) and analysed on real-time qPCR using SYBR^®^ Premix ExTaqII (Takara, Kusatsu, Japan) on an AriaMx Realtime PCR system (Agilent). The results were normalized to *TBP* and *RPS17* housekeeping gene expression levels, using 2^−ΔΔCt^ quantification method.

Next, the relative mtDNA content was calculated as the mean ratio of three mitochondrial genes copy number (*MT-ND1*, *MT-CO1* and *MT-TL1*) to single-copy nuclear genes (*HBB*, *SLCO2B1* and *SERPINA1*, respectively) using the 2^−ΔCt^ quantification method. All primers were listed in the Supplementary Table S1.

### 2.13. Statistical Analysis

Data were expressed as mean or median ± SD. Statistical significance was calculated depending on the experiment design by Student’s *t*-test, one-way analysis of variance (ANOVA), two-way analysis of variance (ANOVA2), or Pearson correlation using Prism software (version 7.0, GraphPad Software, San Diego, CA, USA). Mean differences were considered statistically significant when *p* < 0.05 *, *p* < 0.01 **, *p* < 0.001 ***, *p* < 0.0001 ****.

## 3. Results

### 3.1. Aging Does Not Change the Rate of Senescent Cells in a Culture of Primary Fibroblasts

**Figure 1:**
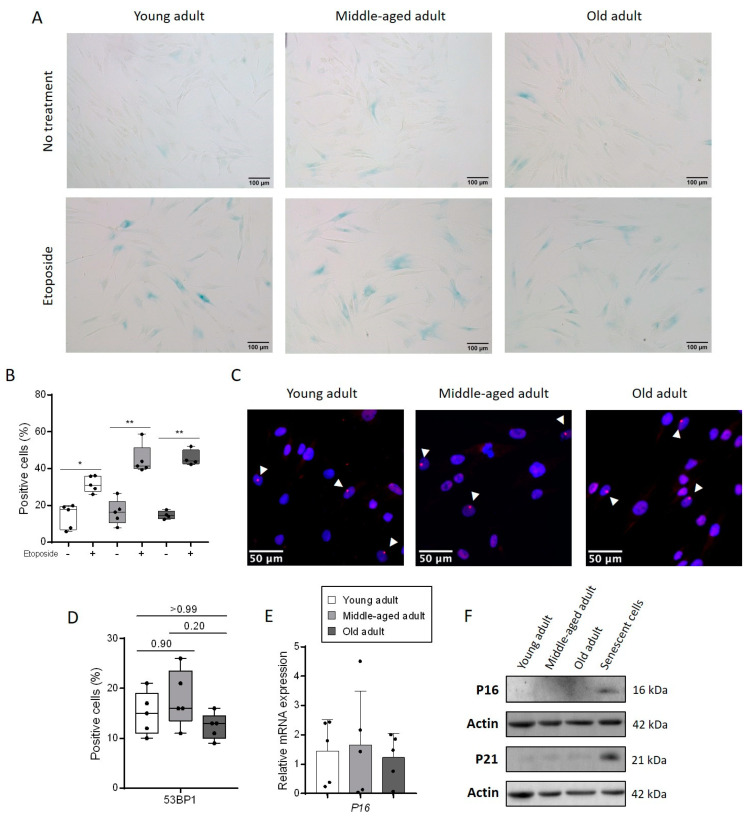
The basal level of senescent cells is equal in the three groups of primary fibroblasts from adults. (**A**) Representative images of SA-βgalactosidase assay for each group of age. 10 µM etoposide treatment was used as a positive control for senescence. (**B**) Quantification of positive cells in SA-βgalactosidase assay with etoposide treatment or not. Five images were analyzed for each primary fibroblasts’ strains in the two conditions (mean ± SD; *n* = 5; * *p* < 0.5, ** *p* < 0.01). Exact *p*-values were determined using a paired Student’s *t*-test for comparison between cells treated with etoposide and cells without treatment. (**C**) Quantification of positive cells for 53BP1 staining. At least 200 cells from three images were analyzed for each primary fibroblast strain (mean ± SD; *n* = 5). (**D**) Representative images of 53BP1 immunostaining for each group of age. White arrows indicate positive cells. (**E**) Relative mRNA expression of P16 in primary fibroblasts. qPCR analysis was normalized to TBP and RPS17 housekeeping genes using the 2^−ΔΔCt^ quantification method (mean ± SD; *n* = 5). (**F**) P16 and P21 protein expressions were evaluated by immunoblotting in the three groups of primary fibroblasts (*n* = 5) and in a senescent positive control (cells treated with etoposide). One representative cell strains from each group of age is represented here. Actin protein expression is used as a loading control.

The increase in the number of senescent cells is one of the main characteristics of aged skin [9,14]. Contradictory data have been published about the impact of chronological aging on the basal senescent profile of dermal fibroblasts in culture [15,16]. Here, we wanted to clarify whether primary fibroblasts isolated from young or aged adult skin and cultured at the same passages (P8) exhibited different rates of senescent cells. We used primary fibroblasts from young, middle-aged and old adult individuals to constitute three age groups. We first analyzed the rate of senescent cells in each group using a SA-βgalactosidase activity test. The treatment with the DNA-damaging reagent etoposide, which is a topoisomerase II inhibitor inducing double-strand breaks, was used as a positive inducer of senescence. We found few positive cells for SA-βgalactosidase activity in the untreated control condition, equivalent for each group, with around 15% of positive fibroblasts (young: 14.0% ± 2.9, middle-aged: 16.3% ± 3.1, old: 14.7 ± 1.1) (Figure 1A,B). After etoposide exposure, the number of SA-βgalactosidase positive fibroblasts doubled in the young fibroblasts (31.6% ± 2.0), while this number tripled in the middle-aged (44.8% ± 3.5) and old (45.6% ± 2.2) fibroblasts. These results suggested that primary fibroblasts isolated from aged donors did not exhibit increased senescence after eight passages. However, the age of the donor may influence the sensitivity of the cells to stress-induced senescence. Genomic instability is one of the main signals for senescence induction. Therefore, an immunostaining of 53BP1 (tumor protein p53 binding protein 1), a protein implicated in DNA double-strand break repair pathway and already described for its implication in premature senescence [17] was performed. The percentage of 53BP1 positive cells was equivalent within the three groups (young: 15.0% ± 1.9, middle-aged: 18.0% ± 2.6, old: 12.4% ± 1.2) (Figure 1C,D) and roughly corresponded to the percentage of positive cells for SA-βgalactosidase. Then, we analyzed P16 transcript expression, a well-recognized senescence marker, and we did not observe a significant difference between the three age groups (Figure 1E). Finally, the protein expression of P16 and P21 was barely observable by a Western blot analysis in all cultures of fibroblast strains (Figure 1F). Taken together, these results demonstrated that the senescence level was equivalent in the cultured fibroblasts regardless of the age of the donor and thus would not contribute to further functional differences, if any, in the following experiments.

### 3.2. Aging Negatively Impacts Primary Fibroblasts Proliferation

**Figure 2:**
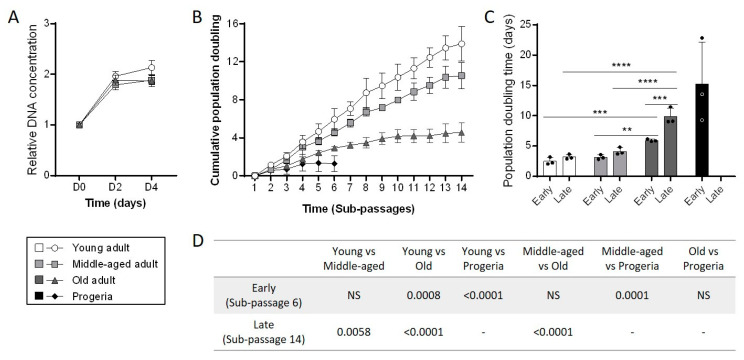
Aging impacts proliferative abilities of primary fibroblasts. (**A**) Short-time cell proliferation of primary fibroblasts obtained from adults according to their age was analyzed by DNA quantification using fluorescent DNA intercalant (CyQuant Kit). (**B**) Cumulative population doubling of primary fibroblasts during 14 sub-passages for cell group (mean ± SD; *n* = 3). (**C**) Population doubling time determined at sub-passage 6 (Early) and sub-passage 14 (Late) for the different primary fibroblasts’ groups (mean ± SD; *n* = 3; * *p* < 0.05, ** *p* < 0.01, *** *p* < 0.001, **** *p* < 0.0001). Exact *p*-values were determined using the Two-way ANOVA and Tukey post hoc tests. (**D**) Statistical results obtained for cumulative population doubling analysis (NS = nonsignificant, *p* > 0.05). Exact *p*-values were determined using the one-way ANOVA and Tukey post hoc tests.

Another characteristic of aged skin is the decline in proliferative cells in the dermis [18]. Therefore, we performed functional assays to evaluate the proliferative capacity of primary fibroblasts in culture. First, we realized a short-term cell proliferation test by measuring DNA concentrations in cultures over four days. We found that the DNA concentrations increased strongly during the first two days in each age category, meaning that fibroblasts rapidly proliferated (Figure 2A). At day four, there was double the amount of DNA than at day 0 for young fibroblasts and slightly lower (×1.8) for middle-aged and old fibroblasts. This difference was not significant and did not lead to a clear distinction between the three groups. Then, we evaluated the long-term cell proliferation capacity by determining the population doubling level over several weeks in culture. At the beginning of the analysis, all fibroblasts were at equivalent passage (7–8) and we identified sub-passages for each passage during the following weeks in culture. The assay was also performed on progeria primary fibroblasts to control mimic accelerated aging [19]. Progeria fibroblasts reached a growth plateau after six sub-passages (sP) only, while all strains of the healthy adult fibroblasts still proliferated (Figure 2B). At this time, a complete population doubling occurred after about 2.5 days for young fibroblasts, 3.1 days for middle-aged fibroblasts, 5.9 days for old fibroblasts and 15.2 days for progeria fibroblasts (Figure 2C). Old fibroblasts were the second group to reach the proliferation plateau after fourteen sub-passages. At this time, a complete population doubling occurred after about 3.2 days for young fibroblasts, 4.1 days for middle-aged fibroblasts and 9.9 days for old fibroblasts (Figure 2C,D). From sP6 to sP14, the population doubling time increased by 28% in young fibroblasts, 32% in middle-aged fibroblasts and 68% in old fibroblasts. This extended time to proliferate over weeks was correlated with the cumulative population doubling (CPD) number in each group. Indeed, from the initial one million cells plated, young fibroblasts were able to undergo 14 CPD, whereas middle-aged fibroblasts completed 10 CPD and old fibroblasts only 4 CPD (Figure 2D). Thus, primary fibroblasts from donors aged over 70 years old seemed to exhaust their proliferative capacity much faster than those from younger donors and even from donors aged between 55 and 65 years old in vitro.

### 3.3. Aging Decreases Clonogenic Abilities of Primary Fibroblasts

**Figure 3:**
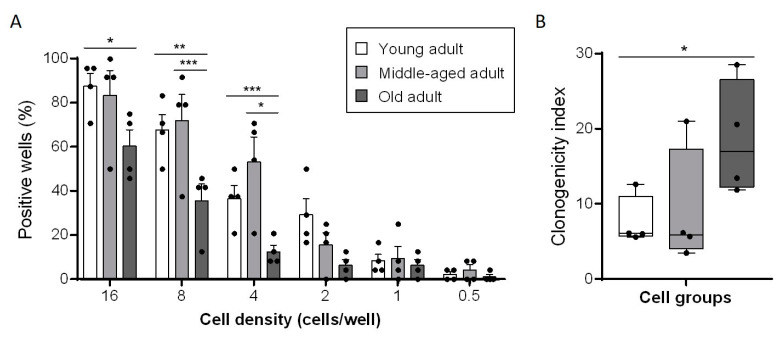
Aging decreases clonogenic abilities of primary fibroblasts. (**A**) Percentage of positive wells obtained for different cell densities with each primary fibroblasts’ strains after 15 days of culture. (mean ± SD; *n* = 4; * *p* < 0.05, ** *p* < 0.01, *** *p* < 0.001). Exact *p*-values were determined using the two-way ANOVA and Tukey post hoc tests. (**B**) Clonogenicity index of primary fibroblasts from each group after 15 days of culture. (mean ± SD; *n* = 4; * *p* < 0.05). Exact *p*-values were determined using the one-way ANOVA and Tukey post hoc tests.

Once the growth plateau was determined by the population-doubling assay, the progenitor cell population in each group and their ability to generate clones was analysed. To evaluate the intrinsic ability of cells to generate clonal expansion, we seeded fibroblasts of the three age groups at six very low densities (16, 8, 4, 2, 1 and 0.5 cells/well) and kept cells in culture for fifteen days. At the end of these two weeks, each of the well-containing cells were classified as positive wells. Each positive well is thus considered as a success and means that among the few fibroblasts at the beginning, there was one cell or more with clonal-expansion capabilities. At 16 cells/well condition, the success of young and middle-aged fibroblasts to generate clones was about 85%, but only 60% for old fibroblasts (Figure 3A). As expected, when the cell-seeding concentration decreased, the number of generated clones decreased too. However, the serial dilutions did not affect each age category in the same way. At eight cells/well, the percentage of positive wells for the young and middle-aged fibroblasts were still around the same (68% and 72%, respectively) whereas the percentage of positive wells for the old fibroblasts dropped to 35%. For each successive dilution, we observed an altered capacity of old fibroblasts to generate clonal expansion, as compared to cells from young or middle-aged fibroblasts. The percentages obtained for old fibroblasts were lower for the four more important cell densities and approximately corresponded to the values obtained with young fibroblasts for the following dilution. To better characterize the intrinsic capacity for clonal expansion in the cell population of each age group, we calculated the clonogenicity index (detailed in the Materials and Methods section). This index was 7.5 for young fibroblasts, 9 for middle-aged fibroblasts and 18.6 for old fibroblasts (Figure 3B). We observed that the clonogenic capacity of young and middle-age fibroblasts were quite similar, whereas the clonogenic capacity of old fibroblasts was significantly altered since double the amount of cells were required to achieve the same efficiency.

### 3.4. Aging Affects Mitochondrial Metabolism in Primary Fibroblasts

**Figure 4:**
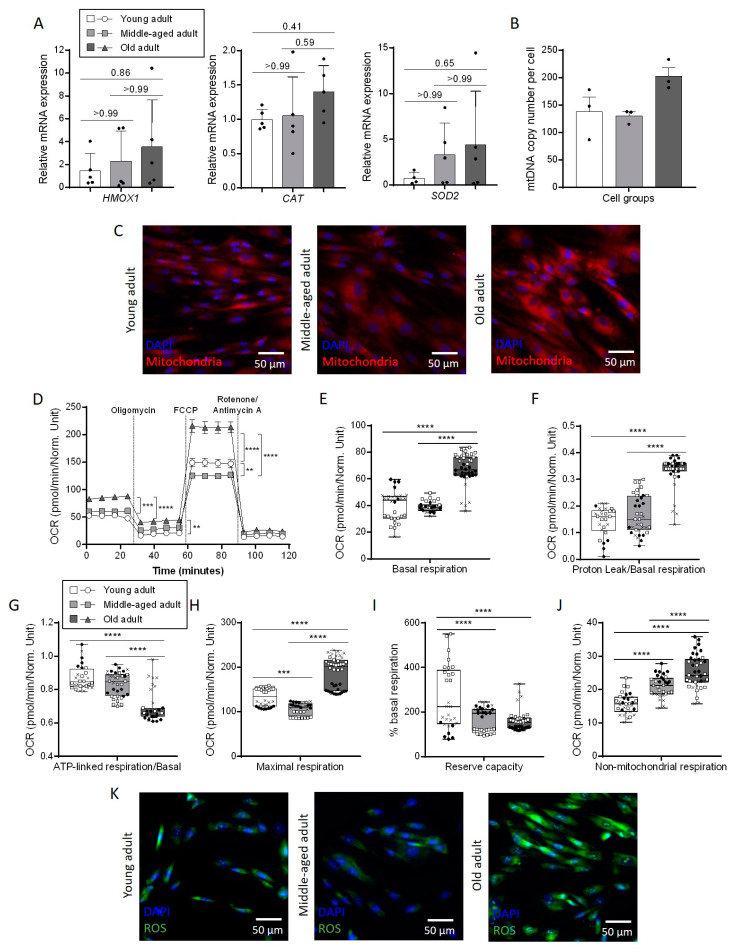
Mitochondrial respiration of primary fibroblasts is affected by aging. (**A**) Relative mRNA expression of HMOX1, CAT and SOD2 in primary fibroblasts. qPCR analysis was normalized to TBP and RPS17 housekeeping genes using the 2^−ΔΔCt^ quantification method (mean ± SD; *n* = 5; * *p* < 0.05). Exact *p*-values were determined using the One-way ANOVA and Tukey post hoc tests. (**B**) Number of mitochondria per cell estimated by qPCR. qPCR analysis was performed by normalizing the relative content of mitochondrial genes (ND1, CO1 and TL1) with the content of nuclear genes (HBB, SLCO2B1 and SERPINA1) using the 2^-ΔCt^ quantification method in the three groups of age (mean ± SD; *n* = 3). (**C**) Representative images of immunofluorescent staining of mitochondria in primary fibroblasts using MitoTracker^TM^. (**D**)Average seahorse profiles representing oxygen consumption ratio measured using Seahorse Bioanalyzer and determined by the sequential additions of 5 µM oligomycin, an ATP synthase inhibitor, 2 µM FCCP, a protonophoric uncoupler, and 1 µM rotenone and antimycin A, two electron transport chain inhibitors in different primary fibroblasts. The major aspects of mitochondrial coupling and respiratory control were basal respiration (**E**), proton leak over basal respiration ratio (**F**), ATP-linked respiration over basal respiration ratio (**G**), maximal respiration (**H**), reserve capacity (**I**) and non-mitochondrial respiration (**J**) (mean ± SD; *n* = 3; * *p* < 0.05, ** *p* < 0.01, *** *p* < 0.001, **** *p* < 0.0001). Each dot represent one measure and we used points, crosses and squares to differentiate technical replicates (between 8 and 16) from biological replicates (*n* = 3). Exact *p*-values were determined using the One-way ANOVA and Tukey post hoc tests. (**K**) Representative images of immunofluorescent staining of ROS in primary fibroblasts using CellROX^TM^.

Fibroblasts are mostly quiescent in healthy skin whereas, during the formation of the granulation tissue in dermal wound, various growth factors released by immune cells stimulate fibroblasts to migrate and to proliferate [20]. This increased proliferative activity requires a high metabolic demand in which mitochondrial metabolism plays a key role. Oxidative stress is directly linked to mitochondrial respiration and the expression of genes encoding for three different enzymes involved in the anti-oxidative response (HMOX1, CAT, SOD2) which progressively increased with aging, even if this tendency was not statistically significant due to a variability in the expression level between cells cultured (Figure 4A). To evaluate the impact of aging on mitochondrial metabolism, the number of mitochondria was determined by qPCR quantification of the mitochondrial genes copy number per single-copy of nuclear genes. This quantification garnered similar results for young (138 mitochondria per cell) and middle-aged fibroblasts (130 mitochondria per cell), whereas it was around 1.47 time higher in old fibroblasts (203 mitochondria per cell) (Figure 4B). This increase is consistent with our fluorescent analysis of mitochondria content (Figure 4C and Supplementary Figure S1A). Indeed, the intensity of the labelling, based on the oxidation of the probe in active mitochondria, was higher in old fibroblasts compared to young and middle-aged fibroblasts. It seems that aging also has an impact on mitochondria in agreement with the notion that mitochondrial dysfunction is a well-known feature of aging. To elaborate, the oxygen consumption rate (OCR) was measured using Seahorse technology for each age group (Figure 4D). At the basal level, OCR for young and middle-aged fibroblasts was around 40 pmol/min (Figure 4E). This rate was amplified for old fibroblasts (approximately 67 pmol/min, equivalent to 168% of the basal respiration in young fibroblasts). This result agreed with the increase in the mitochondria number in this same group described previously. An increase was also observed for proton leak (Figure 4F) whereas ATP-linked respiration decreased (Figure 4G). Relatively to the basal respiration level, these two last parameters were similar within the young and the middle-aged fibroblasts (around 15% for proton leak and 85% for ATP-linked respiration). However, these percentages changed to 35% and 65%, respectively, in the old fibroblasts. This result suggests that the proton permeability of the inner mitochondrial membrane could be affected by age and that the mitochondrial respiration could therefore be less effective. A significant increase in maximal respiration was also observed in old fibroblasts (Figure 4H). However, as compared to basal-respiration variation between old fibroblasts and the two youngest fibroblasts, this increase was not sufficient to reach the same reserve capacity. Indeed, both middle-aged and old fibroblasts had a reserve capacity that decreased by 40% compared to young fibroblasts (Figure 4I). Aging also altered this respiratory parameter. At the end of the analysis, when the respiratory chain is completely inhibited by the different metabolic inhibitors, non-mitochondrial respiration was increased by more than 60% in old fibroblasts compared to young fibroblasts (Figure 4J). Interestingly, with regard to this parameter, the middle-aged group showed an intermediate profile between young and old with an increase of around 30% in terms of OCR compared to young fibroblasts. All these modifications of the mitochondrial respiration were accompanied by an increase in the quantity of ROS, as observed by fluorescence analysis in old fibroblasts compared to young and middle-aged fibroblasts (Figure 4K and Supplementary Figure S1B).

### 3.5. Aging Disturbs Extracellular Matrix Composition Secreted by Primary Fibroblasts

**Figure 5:**
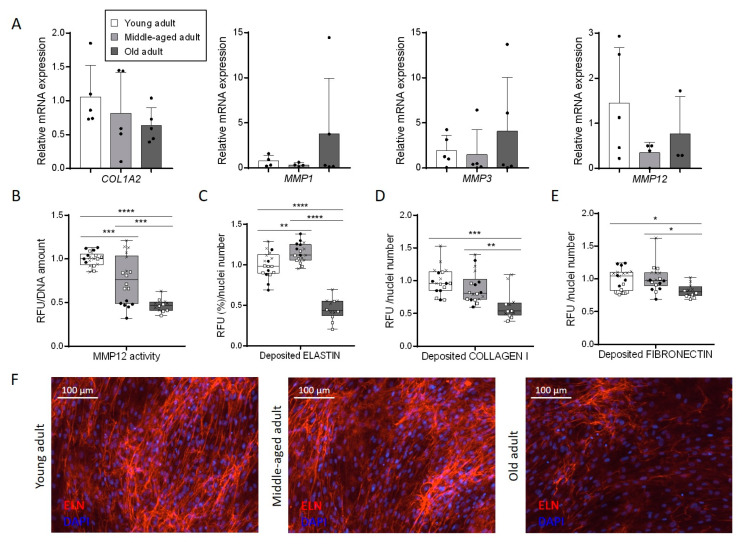
Aging affects ECM production and remodeling in primary fibroblasts. (**A**) Relative mRNA expression of *COL1A2*, *MMP1*, *MMP3* and *MMP12* in primary fibroblasts. qPCR analysis was normalized to *TBP* and *RPS17* housekeeping genes using the 2^−ΔΔCt^ quantification method (mean ± SD; *n* = 5; * *p* < 0.05). Exact *p*-values were determined using the One-way ANOVA and Tukey post hoc tests. (**B**) MMP12 enzymatic activity determined using SensoLyte 520 MMP12 assay kit (mean ± SD; *n* = 3; *** *p* < 0.001, **** *p* < 0.0001). Each dot represents one measure and we used points, crosses and squares to differentiate technical replicates from biological replicates (*n* = 3). Exact *p*-values were determined using the Two-way ANOVA and Tukey post hoc tests. (**C**) Quantification of immunofluorescent staining of ELN expressed as RFU% (relative fluorescence unit vs. total area)/nuclei number ratio in cells. Quantification of immunofluorescent staining of COL1 (**D**) and FN (**E**) in 2D matrix production by primary fibroblasts (mean ± SD; *n* = 3; * *p* < 0.05, ** *p* < 0.01, *** *p* < 0.001, **** *p* < 0.0001). For (**C**–**E**), each dot represents one measure and we used points, crosses and squares to differentiate technical replicates from biological replicates (*n* = 3). Exact *p*-values were determined using the Two-way ANOVA and Tukey post hoc tests. (**F**) Representative images of immunofluorescent staining of ELN in 2D matrix production by primary fibroblasts.

One of the hallmarks of dermal aging is the disorganization of the ECM [21]. Indeed, it is well known that the balance between production and degradation of collagen and elastin fibers is deeply affected in the skin of aged individuals, leading to, in particular, the formation of wrinkles. These marks of age are linked to a lack of the matrix compounds production by fibroblasts and the modulation of the activity of the main enzymes implicated in matrix remodeling. Interestingly, we observed a decrease in the *COL1A2* transcript expression by about 40% and an increase in both *MMP1* (five times more) and *MMP3* (two times more) transcript expressions in the oldest fibroblasts compared to younger fibroblasts (Figure 5A), whereas no significant modulation of *TIMP1* and *TIMP2* transcripts expression was observed (Supplementary Figure S2A). The expression of the *MMP12* transcript, however, seemed to be less doubled in the two older fibroblasts compared to the youngest ones, even if its expression doubled in old fibroblasts compared to middle-aged fibroblasts (Figure 5A). This metalloproteinase is implicated in the remodeling of elastin fibers. We evaluated MMP12 activity and we observed a decreased with aging in our in vitro model. Indeed, a 25% decreased signal was obtained in the middle-aged fibroblasts compared to the young fibroblasts and a decrease of more than 50% was observed in the oldest fibroblasts (Figure 5B). Moreover, the quantity of elastin deposited in the matrix is strongly affected by aging with two times less signal in old fibroblasts compared to young fibroblasts (Figure 5C). This decrease is easily observable on immunofluorescent images (Figure 5F). Concerning the middle-aged fibroblasts, the variation in MMP12 activity described before seems to have no impact on the quantity of deposited elastin in the matrix. Similarly, collagen I and fibronectin synthesis are reduced by, respectively, 38% and 18% in old fibroblasts compared to the quantity measured with young fibroblasts (Figure 5D,E, Supplementary Figure S2B,C). In the case of collagen I, an intermediate profile for the middle-aged fibroblasts was observed, as for the MMP12 activity. Altogether, these data indicate that ECM synthesis and modeling were affected by aging during in vitro culture of fibroblasts. Even if these changes could potentially affect cell migration, we did not observe any difference in the migration abilities of aged fibroblasts in vitro (Supplementary Figure S2D).

## 4. Discussion

One hallmark of chronological skin aging is a slowed proliferation of dermal fibroblasts. In this context, it is expected that primary fibroblasts isolated from people over 70 years old will proliferate less in vitro than fibroblasts from younger persons. In this study, we did not observe any difference in short-time cell proliferation capacity at an equivalent low passage between young, middle-aged and old fibroblasts. However, marked differences appeared when we analyzed proliferation over several weeks. We found that the oldest fibroblasts not only grow slowly, with a higher population doubling time, but also reached a growth plateau faster than younger fibroblasts, probably corresponding to the onset of the replicative senescence. These results seem to adequately mimic one of the aging trait observed in skin tissue [22], in agreement with previous reports on aged fibroblasts [16,23,24,25]. Moreover, the comparison with fibroblasts from patients with Hutchinson–Gilford progeria, a rare genetic condition characterized by an accelerated aging phenotype [19], supports the role of intrinsic aging in the decrease in cell proliferation in vitro. The growth of a cell population mostly depends on the number of progenitor cells that are able to proliferate with high frequency. Hence, aging is also characterized by an exhaustion of progenitor cells [10]. Even if a huge gap of information concerning progenitor cells involved in dermis regeneration exists, several studies showed that some cells in the dermis remain undifferentiated [26]. Moreover, using a clonal analysis of dermal cells, it has been demonstrated that among these cells, some exhibit a potential of differentiation in various mesenchymal lineages and could generate cells with adipogenic, osteogenic or chondrogenic phenotypes. These potential stem cells might represent 0.3% of human dermal foreskin fibroblasts [27]. It remains unclear whether these cells are still present in culture. In this study, we observed an increase in the clonogenic index in vitro according to the age, indicating that the proportion of cells in culture with progenitor features is lower with aging, as it is described in vivo in the human skin. To elaborate, it has been demonstrated that old fibroblasts generate not only less clones, but also smaller clones than young fibroblasts [28]. Collectively, these data support the idea that once in culture, isolated primary fibroblasts display proliferation capacities correlated to the age of the skin donors.

Dermis aging is strongly linked to an ECM deterioration and disorganization, which is primarily caused by a decrease in ECM-protein synthesis by fibroblasts accompanied by an increase in their degradation by metalloproteinases. We observed the specific ECM aging marks in primary fibroblasts in culture. Indeed, in fibroblasts obtained from adults aged over 70 years old, we found significant a decrease in both COL1 and FN synthesis associated with increase in their specific remodeling enzymes, respectively MMP1 [29] and MMP3 [30]. These results are consistent with several previous studies [3,21,28,31]. Whereas MMPs and tissue inhibitors of metalloproteinase (TIMPs) are co-regulated to avoid disproportionate MMP activity in young normal dermis, the overexpression of MMP with aging is no more correctly balanced by a concomitant increase in the TIMP, thus leading to an acceleration of collagen degradation [28,32]. We also observed a strong decrease in both MMP12 activity and deposited elastin quantity in old fibroblasts compared to the youngest fibroblasts. In skin tissue, the main documented cause of elastin degradation is exposition to UV rays, but chronological aging also induces the fragmentation of the elastic fibers network leading to the appearance of fine wrinkles [33]. The morphological changes observed in photo-protected areas from aged individuals compared to young skin tissue are due to an increased vulnerability of elastin fibers to MMP proteolysis with aging [34]. This particular susceptibility could explain why the quantity of deposited elastin is twice lower in old fibroblasts compare to young fibroblasts whereas the activity of MMP12 is also reduced. The observed overexpression of *MMP1*, *MMP3* and *MMP12* is likely linked to an overexpression of *AP-1*, a key transcription factor regulating most MMPs expression, in response to reactive oxygen species (ROS) production [3], another key hallmark of tissue aging [10,35].

More than a hallmark of aging among others, the free radical theory of aging proposes that the ROS-induced deterioration of cellular macromolecules is a primary driving force of aging and a major determinant of lifespan [36]. ROS are highly reactive molecules mainly byproducts of the aerobic respiration. To evaluate the impact of age on aerobic respiration, we analyzed the mitochondrial metabolic activity in fibroblasts and we firstly found an increased number of mitochondria with age in the three groups of fibroblasts. In accordance with our data, a previous study also reported, by quantification of the citrate synthase activity, that mitochondrial mass content progressively increased with age in dermal fibroblasts at early passage (P7) and obtained from donors of young, middle-aged, and old subjects (20, 60, and 90 years) [35]. In addition, another study confirmed this age-related variation of mitochondrial content in the tissue of human skeletal muscle of 27 healthy individuals [37]. Thus, our model of human fibroblasts isolated from different age donors seems to recapitulate a validated feature of human tissue aging. However, the literature is undecided on the effect of aging on mitochondrial metabolism. Indeed, the results are largely dependent on the way of normalization. For instance, a recent study demonstrated that transcriptomic expression of genes related to mitochondrial biogenesis was downregulated with aging and associated with oxidative phosphorylation efficiency decline with age in dermal fibroblasts [38]. However, the quantitation of cellular metabolism was normalized to the protein content of the cells, whereas it has been understood for a long time that aging strongly affects the protein content of fibroblasts with a reduced catabolism of long-lived proteins [39]. In the study of Koziel et al., the authors did not find any significant variation with age in mitochondrial respiration in dermal fibroblasts from human biopsies of three young, three middle-aged and three old individuals, after normalization to citrate synthase activity [35]. In our study, we normalized the data of oxidative metabolism to the number of cells, independently of the number of mitochondria or their enzyme-related activity. Thus, these apparent conflicting data may result from the different methods of normalization. As aging may alter many cell functions, including enzymatic activities, we believe that normalization by the number of cells is preferable. Although we observed a 30% increase in the mitochondrial content in old fibroblasts, the oxygen consumption was increased by 70% in the same group. Thus, the increase in the oxygen consumption with advanced ages is not solely related to the number of mitochondria. However, this apparent increase in mitochondrial respiration in old fibroblasts seemed to be associated with a greater uncoupling of the protons, the coupling efficiency being defined as the proportion of oxygen consumption dedicated to ATP synthesis. Although we observed a coupling efficiency of around 85% in the fibroblasts isolated from both young and middle-aged donors, it dropped to around 65% in the old fibroblasts. Accordingly, the percentage of cells with high mitochondrial membrane potential (ΔΨm) reduces with aging in dermal fibroblasts [35]. The dissipation of the mitochondrial membrane potential provokes the uncoupling of the electron transport through the respiratory chain and increases the proton leak during the phosphorylation reaction for ATP synthesis. These proton and electron leaks are associated with amplified reactive oxygen species (ROS) production and cumulative oxidative damage is responsible for cellular senescence, inflammatory responses and the alteration of ECM dynamics in the skin [40,41]. Accordingly, we also detected a noticeable trend of an increased expression of transcripts for enzymes involved in the regulation of oxidative stress (*HMOX1*, *CAT*, *SOD2*) in primary fibroblasts with aging. Remarkably, a recent study showed that the administration of a moderate mitochondrial complex IV inhibitor in the drinking water of mice every day for sixteen months, reduced ROS production and preserved mitochondrial integrity with aging [42]. Thus, the observed defect in mitochondrial respiration with tissue aging is also conserved in fibroblasts cultured in vitro, independently of their senescence state, and may constitute an excellent model to further explore the mechanisms of aging.

Collectively, our data show that dermal fibroblasts from aged skin retained, even after several subpassages in culture, most of the aging features of the tissue from which they were isolated, especially the disrupted proliferative and metabolic capacities and defective production of ECM. In vitro cultured fibroblasts therefore represent a valuable model to evaluate the impact of chronological aging on cell functions. They also constitute a relevant and useful cellular model for the high-throughput screening of putative anti-aging compounds.

## Data Availability

All data will be made available upon reasonable request.

## Supplementary Materials

The following are available online at https://www.mdpi.com/article/10.3390/cells11050858/s1, Figure S1: Mitochondria and ROS content are modulated by aging; Figure S2: Aging affects ECM production but not the migration abilities; Table S1: List of primers used for qPCR and PCR assays; Table S2: Characteristics of cells used for the study.
