# Supplementary material for: Maintenance of Chronological Aging Features in Culture of Normal Human Dermal Fibroblasts from Old Donors
Source: Cells. 2022 Mar 2;11(5):858. doi: 10.3390/cells11050858 (PMC8909060; doi:10.3390/cells11050858)
Supplement: Supplementary file 1 [file cells-11-00858-s001.zip › cells-1575799-supplementary.pdf]

Supplementary Materials and Methods

Fibronectin and collagen content

Primary antibodies were directed against human fibronectin (rabbit polyclonal antibody, Sigma, France) and human collagen (rabbit polyclonal antibody, Origene, Rockville, MD, USA). Goat anti-rabbit Alexa Fluor 594 (Invitrogen, Waltham, MA, USA) was used as conjugate for elastin. Results were expressed as RFU % (relative fluorescence unit vs. total area) / nuclei number ratio in cells. Goat anti-rabbit Alexa Fluor 488 (Invitrogen, Waltham, MA, USA) was used as conjugate. Fluorescent staining was observed using Cytation 5 cell imaging multi-mode reader and quantified using Gen5 image software (BioTek, Winooski, Vermont, USA).

In vitro wound healing assay

Cells were plated at 38.10^3 cells/cm^2 in 12-well plates. After 48h incubation, cells reached 100% confluency and a wound was produced at the center of the monolayer by gently scraping the cells with a sterile plastic p200 pipette tip to create a wound. After removing incubation medium and washing with PBS, cell cultures were incubated in with DMEM with 2% FBS 1% P/S in order to limit proliferation. All experimental points were observed using light microscopy on Eclipse Ti-E inverted microscope (Nikon) and analysed using ImageJ software.

Table S1. List of primers used for qPCR assays.

Gene	Gene ID	Primer	Primer sequence
TBP	6908	Forward 5′	TCAAACCCAGAATTGTTCTCCTTAT-3′
		Reverse 5′	CCTGAATCCCTTTAGAATAGGGTAGA-3′
RPS17	6218	Forward 5′	CTC TTTTACCAAGGACCCGCC-3′
		Reverse 5′	AGGTTGGACAGACTGCCGAAG-3′
CDKN2A	1029	Forward 5′	GAAGGTCCCTCAGACATCCC-3′
		Reverse 5′	CCCTGTAGGACCTTCGGTGA-3′
HMOX1	3162	Forward 5′	CTTTCAGAAGGGCCAGGTGA-3′
		Reverse 5′	GTAGACAGGGGCGAAGACTG-3′
CAT	847	Forward 5′	TGCGATTCACACCTTTGTGC -3′
		Reverse 5′	TTCATCCAGTGATGAGCGGG -3′
SOD2	6648	Forward 5′	GGCCTACGTGAACAACCTGA-3′
		Reverse 5′	CACGTTTGATGGCTTCCAGC-3′
COL1A2	1278	Forward 5′	TCTGCGACACAAGGAGTCTG-3′
		Reverse 5′	AGCAAAGTTCCCACCGAGAC-3′
MMP1	4312	Forward 5′	ATGAAGCAGCCCAGATGTGGAG-3′
		Reverse 5′	TGGTCCACATCTGCTCTTGGCA-3′
MMP3	4314	Forward 5′	TGAAATTGGCCACTCCCTGG-3′
		Reverse 5′	GGAACCGAGTCAGGTCTGTG-3′
MMP12	4321	Forward 5′	GATGCTGTCACTACCGTGGGAA-3′
		Reverse 5′	CAATGCCAGATGGCAAGGTTGG-3′
HBB	3043	Forward 5′	CATCAAGCGTCCCATAGACTC-3′
		Reverse 5′	ACGTGGATGAAGTTGGTGGT-3′
SLCO2B1	11309	Forward 5′	TGCCGCTCTTCTTTATCGGC-3′
		Reverse 5′	GGCAAGATTTGAGGCAGGGG-3′
SERPINA1	5265	Forward 5′	AAGGTGAGATCACCCTGACG-3′
		Reverse 5′	GTCAGTGAATCACGGGCATC-3′
MTND1	4535	Forward 5′	CAGAGACCAACCGAACCCC-3′
		Reverse 5′	GAAGAATAGGGCGAAGGGGC-3′
MTCO1	4512	Forward 5′	CACACGAGCATATTTCAC-3′
		Reverse 5′	GTACGATGTCTAGTGATGA-3′
MTTL1	4567	Forward 5′	CACCCAAGAACAGGGTTTGT-3′
		Reverse 5′	TGGCCATGGGTATGTTGTTAA-3′

Table S2. Characteristics of cells used for the study.

Goup of age	Cells	Age	Sex	Localisation
Young adults	FS14128	18	Female	Breast
	FS190410	19	Female	Breast
	FS14131	20	Female	Breast
	HNF21053	32	Female	Abdomen
	HNF21046	37	Female	Breast
Middle-aged adults	FS14002	55	Female	Breast
	HNF21044	58	Female	Abdomen
	HNF20072	60	Female	Abdomen
	FS16037	61	Female	Breast
	FS19034	65	Female	Breast
Old adults	HNF21037	72	Female	Abdomen
	HNF73	73	Male	Eyelid
	F103030	74	Female	Abdomen
	HNF210608	75	Female	Back
	HNF78	78	Male	Eyelid

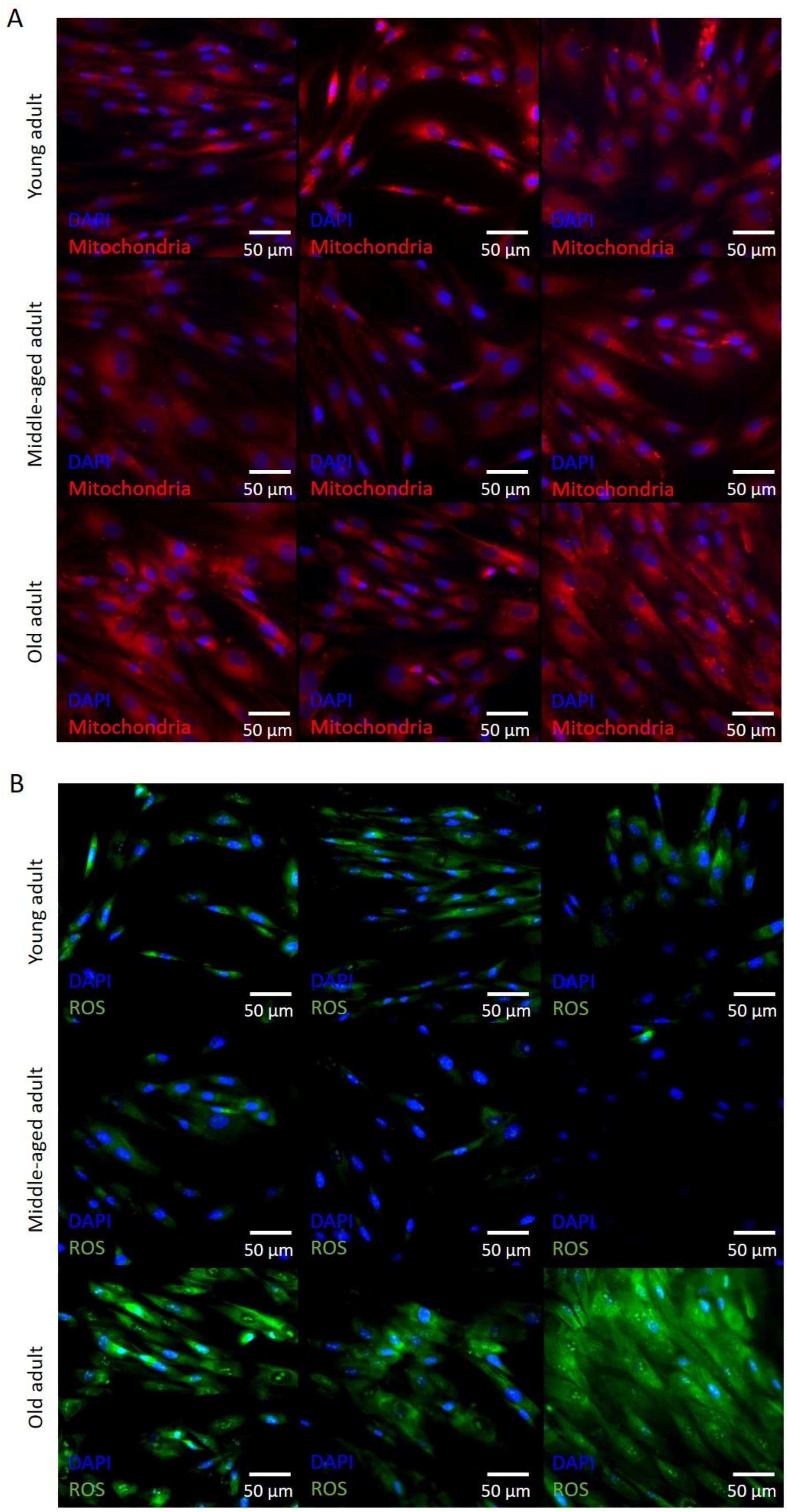

Figure S1. Mitochondria and ROS content are modulated by aging. (**A**) Images of immunofluorescent staining of mitochondria in primary fibroblasts using MitoTracker™. (**B**) Images of immunofluorescent staining of ROS in primary fibroblasts using CellROXTM.

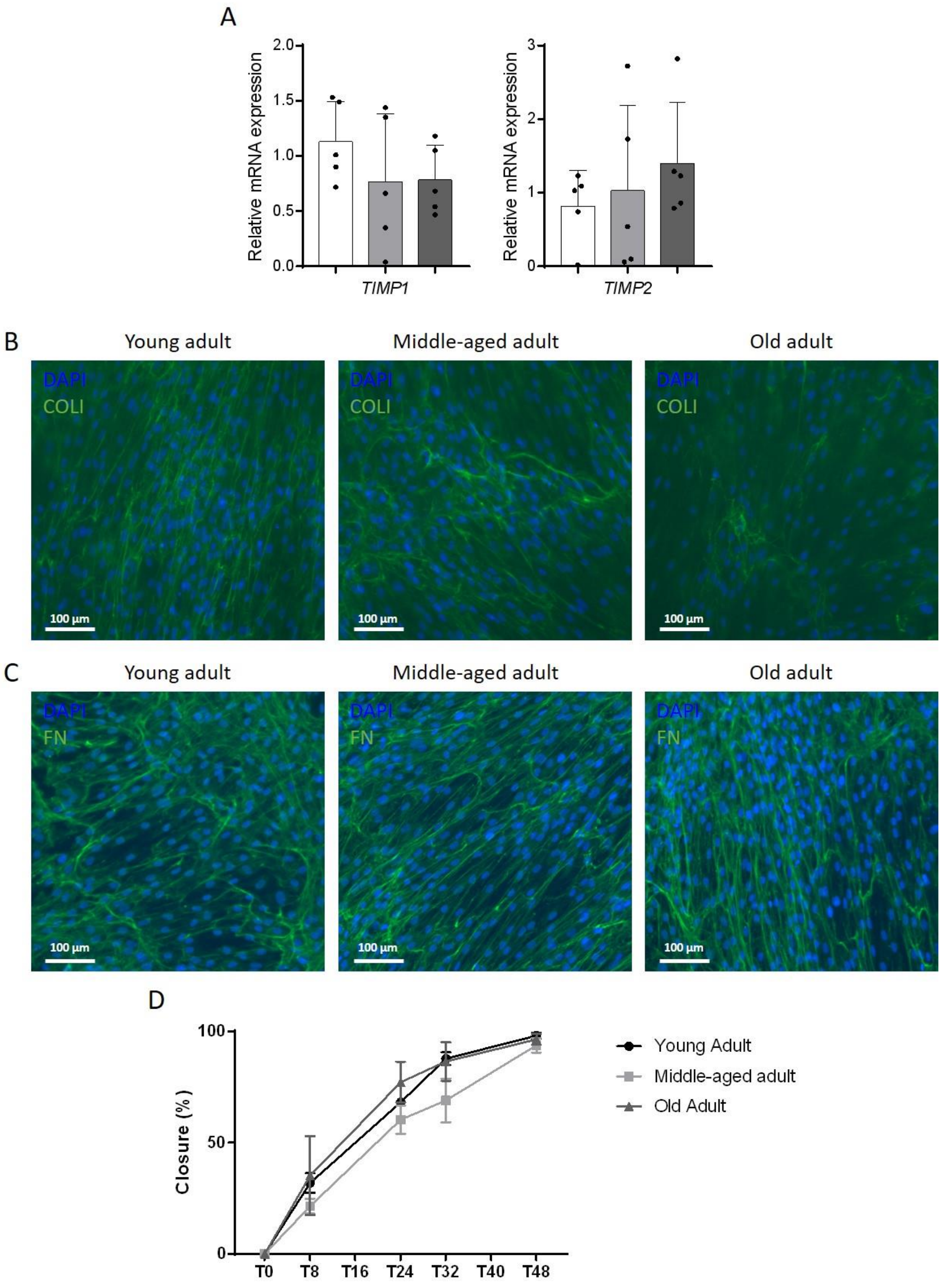

Figure S2. Aging affects ECM production but not the migration abilities. (**A**) Relative mRNA expression of TIMP1 and TIMP2 in primary fibroblasts. qPCR analysis was normalized to TBP and RPS17 housekeeping genes using the 2-ΔΔCt quantification method (mean ± SD; n=5). Exact p-values were determined using the One-way ANOVA and Tukey post-hoc tests. (**B**) Representative images of immunofluorescent staining of COLI and (**C**) FN in 2D matrix production by primary fibroblasts. (**D**) Analysis of the migration rate of primary fibroblasts in a scratch assay.
